# Photonic Hall effect and helical *Zitterbewegung* in a synthetic Weyl system

**DOI:** 10.1038/s41377-019-0160-z

**Published:** 2019-05-29

**Authors:** Weimin Ye, Yachao Liu, Jianlong Liu, Simon A. R. Horsley, Shuangchun Wen, Shuang Zhang

**Affiliations:** 1 College of Advanced Interdisciplinary Studies, National University of Defense Technology, 410073 Changsha, China; 2 Key Laboratory for Micro/Nano Optoelectronic Devices of Ministry of Education, School of Physics and Electronics, Hunan University, 410082 Changsha, China; 3 School of Physics & Astronomy, University of Birmingham, Birmingham, B15 2TT UK; 4 Department of Physics, Harbin Institute of Technology, 150001 Harbin, China; 5 Department of Physics and Astronomy, University of Exeter, Exeter, EX4 4QL UK

**Keywords:** Photonic crystals, Nonlinear optics

## Abstract

Systems supporting Weyl points have gained increasing attention in condensed physics, photonics and acoustics due to their rich physics, such as Fermi arcs and chiral anomalies. Acting as sources or drains of Berry curvature, Weyl points exhibit a singularity of the Berry curvature at their core. It is, therefore, expected that the induced effect of the Berry curvature can be dramatically enhanced in systems supporting Weyl points. In this work, we construct synthetic Weyl points in a photonic crystal that consists of a honeycomb array of coupled rods with slowly varying radii along the direction of propagation. The system possesses photonic Weyl points in the synthetic space of two momenta plus an additional physical parameter with an enhanced Hall effect resulting from the large Berry curvature in the vicinity of the Weyl point. Interestingly, a helical *Zitterbewegung* (ZB) is observed when the wave packet traverses very close to a Weyl point, which is attributed to the contribution of the non-Abelian Berry connection arising from the near degenerate eigenstates.

## Complex light: Crystals could give control

Crystals composed of a honeycomb structure of optically coupled rods with controlled and gradually varying radii could serve as a system to study the phenomenon known as Berry phase effect in the transmission of light. The evidence is presented in theoretical work performed by researchers in China and the UK, led by Shuangchun Wen at Hunan University and Shuang Zhang at the University of Birmingham. Berry connection and Berry curvature involve complex effects imposed upon wave packets of light by regions close to Weyl points. These effects, which involve a phenomenon known as photonic Hall effect, are of interest due to their potential to manipulate light for specialized optical applications. The researchers also discuss issues involved in working toward achieving in practice the effects that their theoretical work reveal, opening the effects to direct study.

## Introduction

Similar to electrons in solid state materials^1^, an optical beam obliquely incident to an interface between two transparent media experiences a spin-dependent transverse shift in the centre of energy (mass) of the reflected/refracted beam. This particular shift is called the spin Hall effect of light (SHEL), which results from the spin−orbit interactions (SOI) or spin−orbit coupling of photons^2–5^. Separate from the light-interface interaction, a pure geometric spin Hall effect^6,7^ is observed for a transmitted optical beam across an oblique polarizer^7^. SHEL has also been studied for structured surfaces, and it was recently shown that a metasurface possessing a linear phase gradient could introduce a giant SHEL for an anomalously refracted beam, even when the light was at a normal incidence^8^. Moreover, analogous to the quantum spin Hall effect of electrons in topological insulators, one-way transport of the spin-valley-locked edge states has been observed in photonic topological insulators^9–12^. With the rapid development in the field of nanophotonics, artificially structured photonic crystals and metamaterials bring about opportunities to manipulate photonic SOI.

Among the reported artificial structures, photonic honeycomb lattices^13–18^ have received considerable attention, in part because of their direct analogue to graphene, where the electron wave has the dispersion of a massless particle close to the Dirac points. Photonic honeycomb lattices provide two new degrees of freedom, pseudospins and valleys, to describe the state of light. For in-plane propagation of light, SHEL was proposed in a photonic analogue of graphene, where the SOI was induced by the splitting between transverse-electric (TE) and transverse-magnetic (TM) optical modes^13^. In a staggered graphene analogue with time-reversal symmetry^14,15^, breaking inversion symmetry can also induce SOI and allow a valley Hall effect of photons^15^. Due to the time-reversal symmetry, there is an opposite transverse shift of the incident optical beam when the in-plane momentum matches different valleys in reciprocal space. Recently, increasing focus has been made on waveguide arrays arranged in a honeycomb lattice, which provides a powerful platform for investigating topological photonics, enabling the realization of photonic Floquet topological insulators^16^, unconventional edge states^17^, and pseudospin-mediated vortex generation^18^.

Berry curvature underlies many interesting phenomena in crystalline systems. It is the counterpart of a magnetic field in momentum space and plays an important role in the motion of a wave packet in both the real space and momentum space^19,20^. It is known, for instance, that the wave packet velocity receives an ‘anomalous’ contribution proportional to the Berry curvature in momentum space^1,19^. In three-dimensional (3D) systems, the sources and drains of Berry curvature are Weyl points, which occur at points of double-degeneracy in the 3D band structure. Weyl points have been found in solid state systems of electrons^21–23^ and 3D photonic crystals^24–26^, magnetized plasma^27^, and photonic metamaterials^28,29^. However, the investigations of Berry curvature effects are not straightforward in photonic systems possessing Weyl points in 3D momentum space [*k*_*x*_, *k*_*y*_, *k*_*z*_]. Since the ‘anomalous’ contribution to the wave packet velocity depends on the product of the time derivative of the wave vector and the momentum space Berry curvature^1,19^, Berry curvature effects cannot be observed in a homogeneous photonic system, which has an invariant wave vector during the propagation. Recently reported synthetic Weyl points^30–32^ provide a new means to construct photonic Weyl points in synthetic 3D space. However, similar to homogeneous Weyl systems in 3D momentum space, a wave packet propagating in the previously reported synthetic Weyl systems^30–32^ has an invariant momentum in the synthetic space and, therefore, cannot interact with the Berry curvature.

## Results

In this Letter we consider a system exhibiting Weyl points in a synthetic 3D space, where two of the axes are momentum coordinates *k*_*x*_ and *k*_*y*_, and the third axis is the physical parameter *η* that adiabatically varies with the spatial coordinate *z*. A wave packet propagating in the *z* direction experiences a variant *η* and, consequently, a *z*-dependent Berry curvature generated by the synthetic Weyl points. It is therefore expected that a Berry curvature-induced Hall effect can be readily observed. To put it simply, as a wave packet moves through a region where the Berry curvature is large, its velocity will be significantly modified from the group velocity, and this effect will be evident as an additional shift in the final position of the packet. Our system consists of an array of evanescently coupled rods tapered along the *z* direction and arranged in a honeycomb array. By slowly varying the diameters of the rods along the direction of propagation (*z*), the two-dimensional (2D) Dirac points in the momentum space can be turned into synthetic Weyl points that, hence, generate a distribution of Berry curvature in the synthetic space. Based on the semi-classic equations of motion for wave packet propagation in the presence of Berry curvature^1,19,20,33,34^, the centre-of-energy velocity of the wave packet contains an ‘anomalous’ contribution proportional to the Berry curvature of the band, and this contribution is responsible for various Hall effects^1,19,33^. Since each Weyl point is a monopole of the Berry curvature in the momentum space, it is expected that a relatively large Hall effect should be observed in its vicinity. In addition to this photonic Hall effect, we also observe an interesting helical *Zitterbewegung* (ZB)^35–38^ arising from the non-Abelian Berry connection close to the Weyl point. That is, the centre of energy of the optical beam exhibits a helical trembling motion around its mean trajectory during the propagation.

**Fig. 1:**
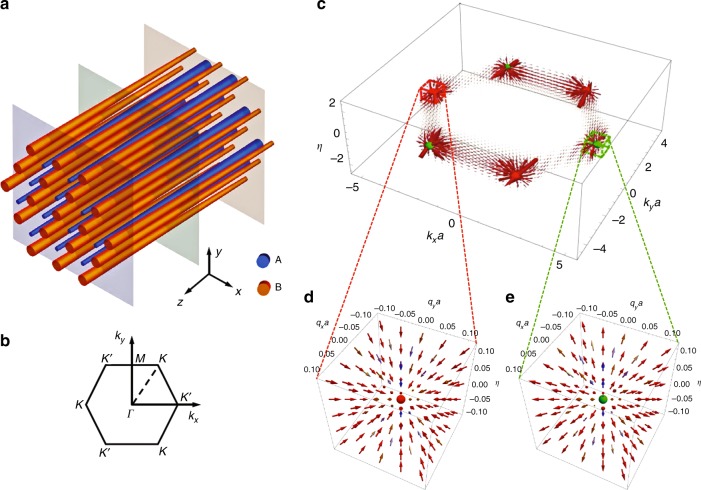
The evanescently coupled rod array arranged in a honeycomb lattice with slowly varying radii along the propagation direction (*z*-direction). **a** The schematic illustration. **b** The first Brillouin zone in the reciprocal space of the honeycomb lattice with the positions of **K** = −$\frac{{4\pi }}{{3a}}$**e**_*x*_ and **K**′ = $\frac{{4\pi }}{{3a}}$**e**_*x*_. **c** The distribution of the Berry curvature of the lower-energy state in the synthetic space [***k***, *ƞ*]. **d**, **e** Local distributions of the Berry curvature close to the two Weyl points

Figure 1a shows our proposed array of dielectric rods with lattice constant *a*. The radii of rods *R*^A^ and *R*^B^ in sub-lattices A and B are designed to vary slowly in the *z* direction. At one end of the rod array, the A lattice has a larger diameter than the B lattice, whereas this is reversed on the opposite end of the waveguide array. Somewhere in the middle of the waveguide array, the A and B lattices have the same size, closing the band gap at this plane. Figure 1b shows the first Brillouin zone of the honeycomb lattice. As will be shown in the following, the Weyl points in the 3D synthetic space are located at points denoted by ***K*** and ***K'***. The propagation of light in the array of rods along *z* axis can be taken as an adiabatic process where the reflection of light in this direction is negligible. Under the tight-binding approximation, the dynamics of the propagation of a Bloch wave with the wave vector (*k*_*x*_, *k*_*y*_) along the rods can be cast into the form of a Schrödinger-type equation for a two-level system (for detailed derivation, see Supplementary information Note 1),

1 $$\begin{array}{*{20}{l}} {i\frac{{\text d}}{{{\text d}Z}}|{\boldsymbol{u}}_{\boldsymbol{k}}\rangle } \hfill & = \hfill & {{\boldsymbol{H}}\left( {{\boldsymbol{k}},\eta } \right)|{\boldsymbol{u}}_{\boldsymbol{k}}\rangle ,} \hfill \\ {{\boldsymbol{H}}\left( {{\boldsymbol{k}},\eta } \right)} \hfill & = \hfill & { - {\text{Re}} \left[ {S({\boldsymbol{k}})} \right]{\mathbf{\sigma }}_x + {\text{Im}} \left[ {S({\boldsymbol{k}})} \right]{\mathbf{\sigma }}_y - \eta {\mathbf{\sigma }}_z} \hfill \end{array}$$

where $\eta = ({\beta^{\mathrm A} - \beta^{\mathrm B}} )/\left(2\kappa \right)$, $S({\boldsymbol{k}}) = 1 + 2\cos \left( \frac{1}{2}k_{x}a \right)e^{i\frac{\sqrt{3}}{2}k_{y}a}$. The normalized coordinate *Z* = $\int \kappa \mathrm {d}z$ is an integral of the effective coupling coefficient *κ* between the nearest-neighbour rods in sub-lattices A and B. *β*^A^ and *β*^B^ are the propagation constants of the fundamental mode supported by isolated rods with radii *R*^A^ and *R*^B^, respectively. Thus, the parameter *η* is *Z* dependent when (*R*^A^−*R*^B^) varies along the propagation direction. Pauli matrices ***σ***_*x*_, ***σ***_*y*_ and ***σ***_*z*_ are defined by the sub-lattice degrees of freedom. The Hamiltonian **H** in Eq. 1 is defined in a synthetic 3D space [***k***, *η*]. Using the generic expression of the Berry curvature of a two-level system^1^, the Berry curvature of the lower-energy state defined in the synthetic space is found to be

2a $$\begin{array}{lll} \Omega _{1} & = & {\Omega _{k_{y}\eta } = \frac{{\sqrt 3 a}}{{2\beta _Z^3}}\cos \left( {\frac{1}{2}k_{x}a} \right)} \\ && \times \left[ {2\cos \left(\frac{1}{2}k_{x}a \right) + \cos \left(\frac{\sqrt 3}{2}k_{y}a \right)} \right] \\ {\beta _Z} & = & \sqrt {\eta ^2 + \left| {S({\boldsymbol{k}})} \right|^2} \end{array}$$

2b $$\begin{array}{lll} {\Omega _{2} = \Omega _{\eta k_x}} & = & \frac{a}{{2\beta _Z^3}}\sin \left( {\frac{1}{2}k_xa} \right)\sin \left( {\frac{{\sqrt 3 }}{2}k_ya} \right)\\ {\Omega _3 = \Omega _{k_xk_y}}& = & \frac{{ - \sqrt 3 a^2}}{{4\beta _Z^3}}\eta \sin \left( {k_xa} \right)\end{array}$$

Figure 1c shows the distribution of the Berry curvature, which becomes singular at the Weyl points located at [±***K***, 0], where the two bands linearly cross each other along all directions in the 3D synthetic space. The Hamiltonian close to the Weyl points can be approximately expressed as

3 $${\mathbf{H}}\left( {\mp \boldsymbol {{K}} + {\mathbf{q}},\eta } \right) = \pm \frac{{\sqrt 3 a}}{2}q_x{\mathbf{\sigma }}_x - \frac{{\sqrt 3 a}}{2}q_y{\mathbf{\sigma }}_y - \eta {\mathbf{\sigma }}_z$$

Furthermore, the Berry curvature (Fig. 1d, e) in its vector form is

4 $${\mathbf{\Omega }}^{(1)}(\mp \boldsymbol {{K}} + {\mathbf{q}},\eta ) = \frac{{ \pm 3a^2}}{{\left( {4\eta ^2 + 3q^2a^2} \right)^{3/2}}}\left( {q_x,q_y,\eta } \right)$$

Due to the presence of time-reversal symmetry, the Berry curvature has an opposite sign at valleys ***K*** and ***K***′. It is straightforward to show that by integrating the Berry curvature in Eq. 4 over an equi-energy surface enclosing a single Weyl point, one can obtain a value of ±2π corresponding to a quantized Chern number of ±1.

Similar to a wave packet propagating in the waveguide array, the equation of the motion for the centre-of-energy coordinate of the Bloch wave ***X***_*C****k***_ is given by (for detailed derivation, see Supplementary information Note 2)

5 $$\frac{{{\text d}{\boldsymbol{X}}_{{\mathrm{C}}{\boldsymbol{k}}}}}{{{\text d}Z}} = \frac{{\langle {\boldsymbol{u}}_{\boldsymbol{k}}|\partial _{\boldsymbol{k}}{\boldsymbol{H}}({\boldsymbol{k}},\eta )|{\boldsymbol{u}}_{\boldsymbol{k}}\rangle }}{{\langle {\boldsymbol{u}}_{\boldsymbol{k}}|{\boldsymbol{u}}_{\boldsymbol{k}}\rangle }} + \left( {{\boldsymbol{X}}_0^{\text B} - {\boldsymbol{X}}_0^{\text A}} \right)\frac{{\langle {\boldsymbol{u}}_{\boldsymbol{k}}|{\mathbf{\Xi }}({\boldsymbol{k}})|{\boldsymbol{u}}_{\boldsymbol{k}}\rangle }}{{\langle {\boldsymbol{u}}_{\boldsymbol{k}}|{\boldsymbol{u}}_{\boldsymbol{k}}\rangle }}$$

in which ${\mathbf{\Xi }}({\boldsymbol{k}}) = {\text{Im}} \left[ {S({\boldsymbol{k}})} \right]{\mathbf{\sigma }}_x + {\text{Re}} \left[ {S({\boldsymbol{k}})} \right]{\mathbf{\sigma }}_y$. ${\boldsymbol{X}}_0^{\text A}$ and ${\boldsymbol{X}}_0^{\text B}$ are, respectively, the relative positions of rods located at sub-lattices A and B within one unit cell. The first term on the right-hand side describes the motion of the Bloch wave determined by the Hamiltonian. The second term is the velocity of the centre of energy due to the transition between sub-lattices A and B within each unit cell of the honeycomb lattice. The Bloch wave can be expressed as a superposition of the lower- and upper-energy states {***Ψ***_1_, ***Ψ***_2_} of the Hamiltonian in Eq. 1 with the eigenvalues {−*β*_Z_, *β*_Z_}, i.e., $|{\boldsymbol{u}}_{\mathbf{k}}\rangle = [|{\boldsymbol{\psi }}_1\rangle ,|{\boldsymbol{\psi }}_2\rangle ]{\boldsymbol{\rho }},{\boldsymbol{\rho }} = [b_1,b_2]^T$. The column vector ***ρ*** describes the coefficients of the eigenstates in the Bloch wave. Thus, the Schrödinger-type equation (Eq. 1) and the centre-of-energy velocity of the Bloch wave (Eq. 5) can be rewritten as (for detailed derivation, see Supplementary information Note 3)

6a $$i\frac{{\text d}}{{{\text d}Z}}{\mathbf{\rho }} = \left[ {{\bar{\boldsymbol H}}_{\boldsymbol{k}} - {\mathbf{\Lambda }}^{(\eta )}\eta {\prime}} \right]{\boldsymbol{\rho }}$$

6b $$\frac{\text {d}}{{\text {d}Z}}\left[ {{\boldsymbol{X}}_{{{C}}{\boldsymbol{k}}} - \left\langle {{\mathbf{\Lambda }}^{({\boldsymbol{k}})}} \right\rangle } \right] = \left\langle {\partial _{\boldsymbol{k}}{\bar{\boldsymbol H}}_{\boldsymbol{k}}} \right\rangle - \left\langle {{\mathbf{\Omega }}^{({\boldsymbol{k}}\eta )}} \right\rangle \eta \prime + {\boldsymbol{V}}_{{\text{PT}}} - \left\langle {\frac{\text {d}}{{\text {d}Z}}{\mathbf{\Lambda }}^{({\boldsymbol{k}})}} \right\rangle$$

where $\eta \prime = {\text {d}}\eta /{\text {d}}Z,{\bar{\boldsymbol H}}_{\boldsymbol{k}} = {\text{diag}}[ - \beta _Z,\beta _Z]$. The Berry connections ***Λ***^(***k***)^ and ***Λ***^(*η*)^ defined in the synthetic 3D space are 2 × 2 matrices with non-zero elements given by ${\mathbf{\Lambda }}_{12}^{({\boldsymbol{k}})} = i\langle {\boldsymbol{\psi }}_1|\partial _{\boldsymbol{k}}{\boldsymbol{\psi }}_2\rangle$ and ${\mathbf{\Lambda }}_{12}^{(\eta )} = i\langle {\boldsymbol{\psi }}_1|\partial _\eta {\boldsymbol{\psi }}_2\rangle$. In Eq. 6b, $\left\langle \circ \right\rangle$ refers to the state average, e.g., $\left\langle {{\mathbf{\Lambda }}^{({\boldsymbol{k}})}} \right\rangle = {\boldsymbol{\rho }}^ + {\mathbf{\Lambda }}^{({\boldsymbol{k}})}{\boldsymbol{\rho }}$. The right-hand side of Eq. 6b consists of four velocity terms. The first term is the average group velocity. The second term is the anomalous velocity^1^, which is proportional to the Berry curvature ${\mathbf{\Omega }}^{({\boldsymbol{k}}\eta )} = i[ {{\mathbf{\Lambda }}^{({\boldsymbol{k}})}{\mathbf{\Lambda }}^{(\eta )} - {\mathbf{\Lambda }}^{(\eta )}{\mathbf{\Lambda }}^{({\boldsymbol{k}})}} ]$ in the synthetic space [${\mathbf{\Omega }}_{11}^{(k_y\eta )} = \Omega _1$ in Eq. 2a and ${\mathbf{\Omega }}_{11}^{(k_x\eta )} = - \Omega _2,{\mathbf{\Omega }}_{11}^{(k_xk_y)} = {\mathbf{\Omega}} _3$ in Eq. 2b]. Close to the Weyl points in the synthetic space studied here, this anomalous velocity is perpendicular to the incident plane and therefore leads to the photonic Hall effect. The third term ${\boldsymbol{V}}_{{\text{PT}}} = \left( {{\boldsymbol{X}}_0^{\text{B}} - {\boldsymbol{X}}_0^{\text{A}}} \right)\left\langle {{\mathbf{\Xi }}({\boldsymbol{k}})} \right\rangle$ is the second term in Eq. 5. Because lattices A and B correspond to the two different pseudospin states in the honeycomb system, this velocity is therefore named the pseudospin transition velocity (PTV). The fourth term corresponds to the contribution from the gradient of Berry connections ***Λ***^(***k***)^ in *Z*. Another contribution to the motion comes from the displacement $\left\langle {{\mathbf{\Lambda }}^{({\boldsymbol{k}})}} \right\rangle$ on the left-hand side of the equation. The displacement induced by the non-Abelian Berry connection (with nonvanishing off-diagonal elements) plays an essential role in generating the ZB effect, where the optical beam features a trembling motion around its mean trajectory, which is the photonic analogue of the behaviour of a free-electron wave packet described by the Dirac equation^35–37^. Note that although the Berry connection ***Λ***^(***k***)^, the Berry curvature ***Ω***^(***k****η*)^ and the average group velocity $\partial _{\boldsymbol{k}}{\bar{\boldsymbol H}}_{\boldsymbol{k}}$ are individually gauge dependent, their overall contribution in Eq. 6b is gauge independent. Thus, the centre-of-energy velocity is non-Abelian gauge invariant. During the adiabatic evolution, off-diagonal elements of the Berry connection with nondegenerate modes are usually negligible. An non-Abelian Berry connection only appears in the synthetic space with degenerate or near degenerate states, such as in the vicinity of a Weyl point.

**Fig. 2:**
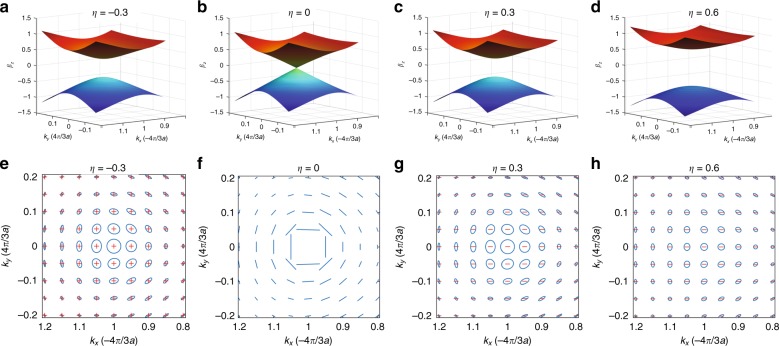
Band structure and distribution of the trajectories of the ZB motions projected to the *x−y* plane in the momentum space (*k*_*x*_, *k*_*y*_) near the K point. **a**–**d** Band structures with parameter *η* equal to −0.3, 0, 0.3 and 0.6, respectively. **e**–**h** Corresponding distributions of the trajectories of the ZB motions. The ellipse (circle) denoted by +(−) means the trajectory of the ZB is a counter-clockwise (clockwise) ellipse (circle) along the propagation direction. The geometric sizes of the line, ellipses and circles represent the relative amplitudes of the ZB with different [*k*_*x*_, *k*_*y*_, *η*]

To look into the dependence of ZB motion over the detailed form of the band structure, we consider a rod array with a uniform radius of rods along the propagation direction of light, that is *η*′ = 0. A simple expression of the centre-of-energy position^38^ can obtained from Eq. 6b

7 $$\begin{array}{l}{\boldsymbol{X}}_{{{C}}{\mathbf{k}}} = b_1^ \ast b_2\left[ {e^{i2\beta _Z\left( {Z_0 - Z} \right)} - 1} \right]\bar \Lambda _{12}^{({\boldsymbol{k}})} + {\text{c}}{.}{\text{c}}{.}\\ \qquad\quad+ \left( {\left| {b_2} \right|^2 - \left| {b_1} \right|^2} \right)\left( {\partial _{\boldsymbol{k}}\beta _Z} \right)\left( {Z - Z_0} \right)\end{array}$$

where $\bar {\Lambda} _{12}^{(\boldsymbol{k})} = \Lambda _{12}^{(\boldsymbol{k})} - {\mathbf{e}}_{y}aS(\boldsymbol{k})/( 2\sqrt 3 \beta _Z)$, and c.c. denotes the complex conjugate. Equation 7 shows that the ZB motion (first term on the right-hand side) can occur at a frequency equal to the band gap 2*β*_z_ when the incident wave is in the superposed state. It is worth noting that the ZB motion is proportional to the complex vector $\bar {\Lambda} _{12}^{(\boldsymbol{k})}$ [≈$\it{\Lambda} _{12}^{(\boldsymbol{k})}$ in the vicinity of **K** point because *S*(**K**) = 0], which directly displays the trajectory of the ZB motion projected onto the *x−y* plane. Figure 2 shows that the ZB motion depends on both the momentum relative to **K** point and the width of the band gap. In the case that the band gap closes (*η* = 0, Fig. 2b), the ZB motion is linear, with azimuthally oriented linear motion relative to the **K** point (Fig. 2f). Exactly at the **K** point, the ZB disappears due to the degeneracies of the two pseudospin eigenstates. Interestingly, for non-zero *η* (Fig. 2a, c, d), the ZB motion is elliptical, having an increasing amplitude and a more circular trajectory when the momentum is closer to the **K** point (Fig. 2e, g, h). Importantly, the direction of the motion (clockwise or counter-clockwise) depends on the sign of *η*. Away from the Weyl point (double degenerate) when increasing the parameter *η* or the distance from the **K** point, the amplitude of the ZB motion decreases because the non-Abelian Berry connection decreases with the widened band gap.

**Fig. 3:**
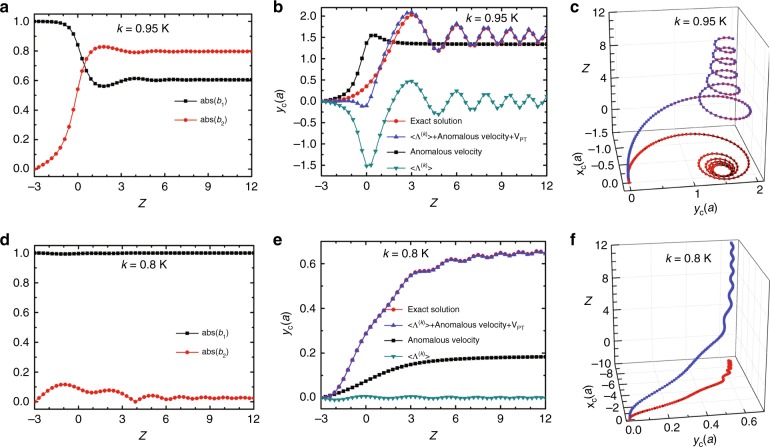
Evolution of the Bloch wave propagating in the array of rods with varying radii along the propagation direction with *ƞ* = 0.23 *Z*. For the incident Bloch wave with a wave vector ***k*** equal to 0.95**K** in the lower-energy state, **a** shows the calculated two mode coefficients by solving Eq. 6a. **b** shows the exact centre-of-energy transverse shift (Hall shift, denoted by circles) obtained by solving Eq. 6b, and the calculated centre-of-energy transverse shifts induced by the combined contribution of $\langle {{\mathbf{\Lambda }}^{({\boldsymbol{k}})}} \rangle$ and anomalous velocity and ***V***_PT_ (denoted by upper triangles), and only the contribution of the anomalous velocity (denoted by squares), and only the contribution of the displacement $\langle {{\mathbf{\Lambda }}^{({\boldsymbol{k}})}} \rangle$ (denoted by lower triangles), respectively. **c** Spatial trajectory of the centre of the energy of the wave packet. **d**–**f** Corresponding results of the incident Bloch wave with the wave vector ***k*** equal to 0.8 **K**

Next, we consider a variation of the radii of rods along the propagation direction, with a linear dependence *ƞ* = 0.23*Z*. For a Bloch wave in the lower-energy state with wave vector ***k*** = 0.95 **K** at the incident point *Z*_0_ = −3, Fig. 3a shows the evolution of the two mode coefficients during the propagation (Eq. 6a). Close to the location of the Weyl point (*Z* = 0) where two eigenmodes are nearly degenerate, the two coefficients experience rapid changes, and the magnitude of the non-Abelian Berry connection **Λ**^*(η)*^ is at its maximum. The coefficient of the upper-energy state (*b*_2_) significantly increases and becomes greater than that of lower-energy state (*b*_1_). When *Z* is greater than 2, the Bloch wave turns into a superposed state with the two relatively stable mode coefficients. Figure 3b shows the evolution of centre-of-energy shift in the *y* direction (Hall shift) induced by the displacement $\langle {{\mathbf{\Lambda }}^{({\boldsymbol{k}})}} \rangle$, the anomalous velocity, and the combined contribution of $\langle {{\mathbf{\Lambda }}^{({\boldsymbol{k}})}}\rangle$, the anomalous velocity, and ***V***_PT_, respectively. The combined contribution (the last one) agrees well with the exact Hall shift calculated using Eq. 6b. It follows that the ZB motion and the transverse trajectory of the centre-of-energy of the Bloch wave are mainly determined by $\langle {{\mathbf{\Lambda }}^{({\boldsymbol{k}})}} \rangle$ and the anomalous velocity, whereas ***V***_PT_ is relatively small. Away from the Weyl point when *Z* is greater than 3, the anomalous velocity becomes very small due to the nearly vanishing Berry curvature. Similar with the array having a uniform radius in Fig. 2, the motion of the Bloch wave in the real space exhibits a spiral trajectory but has a decreased diameter during the propagation away from the Weyl point (Fig. 3c). On the other hand, a Bloch wave in the lower-energy state with a wave vector ***k*** = 0.8 **K** (which is relatively far away from the location of the Weyl point), stays mainly in the low-energy state with a very small upper-energy state coefficient during the propagation (Fig. 3d). This is due to the nearly vanishing Berry connection and Berry curvature (Fig. 1c). The Hall shift induced by the displacement $\langle {{\mathbf{\Lambda }}^{({\boldsymbol{k}})}} \rangle$ is approximately zero. Interestingly, the pseudospin transition velocity ***V***_PT_ plays a leading role in the Hall shift (Fig. 3e). A small wiggling feature at a large *Z* is a result of the contribution by ***V***_PT_ and is shown in Fig. 3f.

**Fig. 4:**
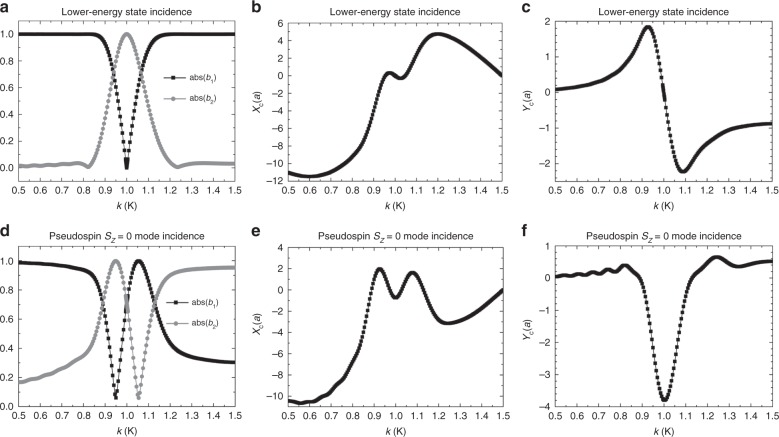
Behaviour of the output waves for Bloch waves incident to the array of rods array with different in-plane components of *k* in the *x* direction. The rod array is same as that shown in Fig. 2, with a fixed normalized length of 15. For the incident Bloch wave in the lower-energy state at the incident point *Z*_0_ = −3, **a** shows the two calculated mode coefficients of the output Bloch wave; **b**, **c** present *x*- and *y*-directional centre-of-mass shifts; and **d**−**f** show the corresponding results of the incident Bloch wave with the pseudospin *S*_*z*_ = 0, that is, $|{\boldsymbol{u}}_{\boldsymbol{k}}(Z_0)\rangle = [1\quad 1]^T/\sqrt 2$ in Eq. 1

Furthermore, we investigate the behaviour of the output waves when a Bloch wave is incident onto the rod array with varying in-plane wave vectors in the *x* direction. Here the *y*-direction centre-of-energy shift corresponds to the Hall shift. The *Z* coordinate of the input and output interfaces are fixed at −3 and 12, respectively, which guarantees that the Bloch wave will pass through the location of the Weyl point (*Z* = 0) during its propagation. As the Hall shift depends on the initial states, we studied the momentum dependence for two different initial states: the lower-energy state and the pseudospin *S*_*z*_ = 0 state (equal mixing between sub-lattices A and B). For an incident Bloch wave in the lower-energy state, only when the wave vector is in the vicinity of the **K** point, it can be converted into a combination of states at the output facet (Fig. 4a) due to the effect of the non-Abelian Berry connection. However, when the incident Bloch wave with wave vector exactly matching the **K** point, its energy is only located at the sub-lattice B during the propagation due to the conservation of the *z* component of the pseudospins. As a result, *x*- and *y*-directional shifts of its centre of energy are zero (Fig. 4b, c). In the vicinity of the **K** point, the Hall shift (Fig. 4c) displays a Fano-like line shape. On the other hand, for an incident Bloch wave in the pseudospin *S*_*z*_ = 0 state, the mode coefficient and the lateral shifts of the beam exhibit quite different line shapes from that of the lower-energy initial state (Fig. 4a–c) in the vicinity of the **K** point, as shown in Fig. 4d–f. A clear enhancement of the Hall shift at the **K** point is evident in Fig. 4f. Since the Hall shift in our system originates from the pseudospin−orbit interactions (Eq. 1), different pseudospins lead to different wave motion.

## Discussion

To confirm the above results, we simulate the propagation of Gaussian beam in the rod array (for details, see Supplementary information Note 4). A hexagonal array (Fig. 1a) with 50 rods arranged on each side (14,554 rods in total) is used in the calculation. An incident Gaussian beam with a centre of energy ***X***_0_, central wave vector ***k***_C_, and beam waist radius *W* is selected to be in the lower-energy state of the Hamiltonian ***H***(***k***_C_, *η*(*Z*_0_)) at the incident position. We calculate the centre-of-energy shifts and 3D trajectories of two Gaussian beams with parameters (***X***_**0**_, ***k***_C_, *W*) equal to (0, 0.8**K**, 50/|**K**|) and (0, 0.95**K**, 50/|**K**|), and find that both trajectories agree well with those of the Bloch wave obtained by solving Eqs. 6a and 6b, as detailed in the Supplementary information. Thus, the propagation of Gaussian beams with relatively large waist radii are well described by Bloch waves.

In conclusion, we have proposed and demonstrated that a coupled rod array arranged in a honeycomb lattice with variant radii possesses photonic Weyl points in a 3D synthetic space, which become Dirac points when projected down to a 2D momentum space. The advantage of our system is obvious for probing the Berry phase effects that arise from Weyl points. Due to the presence of a local Berry curvature in the synthetic space, the wave packets experience an anomalous velocity in the vicinity of Weyl points, which leads to an enhanced Hall effect. Furthermore, because the eigenstates of the system at the Weyl point are double-degenerate, a non-Abelian Berry connection appears in the vicinity of the Weyl point, which leads to a helical ZB effect. Therefore, our system provides a powerful platform for studying the effects of the Berry phase on the dynamics of photonic wave packets.

## Materials and methods

We discuss the practical realization of the rod array necessary to reproduce the above findings. Similar to the reported photonic graphene analogues fabricated via femtosecond direct laser writing in fused silica^17,39^, we select the refractive index of the rods Δ*n* = 2 × 10^−4^ greater than the silica background. At a wavelength of 488 nm, the radii *R* of rods in sub-lattice A (B) linearly increase (decrease) from 3.5 μm (4.5 μm) to 6 μm (2 μm) in *z* direction at the very small ratio Δ*R*/Δ Z= 0.5 × 10^−4^, which corresponds to (*β*^A^ − *β*^B^)/2 increasing from −2 to 7 cm^−1^. Thus, the total length of the silica rods array is approximately 5 cm. Furthermore, with the lattice constant *a* equal to 21 μm, the averaged effective coupling coefficient *κ* is approximately 2.9 cm^−1^ (for details, see Supplementary information Note 5). It is worth noting that the incident Bloch wave with the pseudospin *S*_*z*_ = 0 is equivalent to an incident Gaussian wave. So, by gradually varying the incident angle of the Gaussian beam, we can measure the relative displacements between the centres of the input and output beams using a quadrant detector^8^. Furthermore, the helical ZB can be observed by measuring the centre-of-energy displacement of the output beam through rod arrays of different lengths.

## Supplementary information

Supplementary Material
