# Supplementary material for: Photonic Hall effect and helical Zitterbewegung in a synthetic Weyl system
Source: Light Sci Appl. 2019 May 29;8:49. doi: 10.1038/s41377-019-0160-z (PMC6538614; doi:10.1038/s41377-019-0160-z)
Supplement: Supplementary file 1 — Supplementary Material [file 41377_2019_160_MOESM1_ESM.docx]

**Supplementary Information**

**Photonic Hall effect and helical *Zitterbewegung* in a synthetic Weyl system**

Weimin Ye^1^, Yachao Liu^2, 3^, Jianlong Liu^4^, Simon A. R. Horsley^5^, Shuangchun Wen^2#^, and Shuang Zhang^3*^

1. *College of Advanced Interdisciplinary Studies, National University of Defense Technology, Changsha, 410073, China*
2. *Key Laboratory for Micro/Nano Optoelectronic Devices of Ministry of Education, School of Physics and Electronics, Hunan University, Changsha, 410082, China*
3. *School of Physics & Astronomy, University of Birmingham, Birmingham, B15 2TT, UK*
4. *Department of Physics, Harbin Institute of Technology, Harbin 150001, China*
5. *Department of Physics and Astronomy, University of Exeter, EX4 4QL, England, UK*

^#^scwen@hnu.edu.cn

*s.zhang@bham.ac.uk

**Note 1. The detailed derivation of the dynamics equations of the propagation of Bloch wave along the rods array [Eq. 1 in the main text]**

For the sake of simplicity, we consider the coupling between two rods with slowly varying radii *R*^A^(*z*) and *R*^B^(*z*) satisfying Eq. s1a. Neglecting the reflection of light propagating along the rods, under the tight-binding approximation, the electric fields in the two rods can be written as

$\left|dR^{A(B)}(z)/dz\right|\ll1$, $\left|d\bar{\chi}^{A(B)}(z)/dz\right|\ll\beta^{A(B)}\left|\bar{\chi}^{A(B)}(z)\right|$ (s1a)

$\mathbf{E}=e^{-i\omega t}\left[\bar{\chi}^{A}(z)e^{i\int\beta^{A}dz}\mathbf{E}^{A}\left(R^{A}(z),\mathbf{X}_{\perp}-\mathbf{X}_{0}^{A}\right)+\bar{\chi}^{B}(z)e^{i\int\beta^{B}dz}\mathbf{E}^{B}\left(R^{B}(z),\mathbf{X}_{\perp}-\mathbf{X}_{0}^{B}\right)\right]$, (s1b)

where, $\bar{\chi}^{A}$and$\bar{\chi}^{B}$are the slowly varying amplitudes of fundamental modes satisfying Eq. s1a. In Eq. s1b, $\mathbf{X}_{0}^{A}$ and $\mathbf{X}_{0}^{B}$ are the relative positions of rods A and B in one unit cell, respectively. ***E***^A(B)^ and *β*^A(B)^ are the electric field and the propagation constant of the fundamental mode supported by an isolated rod with the fixed radius *R*^A(B)^ , respectively, and satisfy

$\nabla\times\nabla\times\left[e^{i\beta^{A(B)}z-i\omega t}\mathbf{E}^{A(B)}\right]=\omega^{2}\mu_{0}\varepsilon^{A(B)}\left[e^{i\beta^{A(B)}z-i\omega t}\mathbf{E}^{A(B)}\right]$, (s2a)

. (s2b)

Where, *ɛ*_1_ and *ɛ*_2_ are the dielectric constants of the isolated rod and that of background medium, respectively. The electric field in the system of two rods (Eq. s1b) satisfies the same form of equation as for the isolated rod

$\nabla\times\left(\nabla\times\mathbf{E}\right)=\omega^{2}\mu_{0}\varepsilon\mathbf{E}$, (s3a)

. (s3b)

Under the first order approximation, combing Eq. s1b with Eq. s3a, we have

$\begin{matrix} \nabla\times\left(\nabla\times\mathbf{E}\right)=e^{-i\omega t}\left\{\begin{matrix} \bar{\chi}^{A}(z)\left(i\omega\mu_{0}\right)\left(-i\omega\varepsilon^{A}\right)e^{i\int\beta^{A}dz}\mathbf{E}^{A}+\bar{\chi}^{B}(z)\left(i\omega\mu_{0}\right)\left(-i\omega\varepsilon^{B}\right)e^{i\int\beta^{B}dz}\mathbf{E}^{B} \\ +\left(\partial_{z}\bar{\chi}^{A}\right)\left\{\nabla\times\left(\mathbf{e}_{z}\times e^{i\int\beta^{A}dz}\mathbf{E}^{A}\right)+\left(i\omega\mu_{0}\right)\mathbf{e}_{z}\times e^{i\int\beta^{A}dz}\mathbf{H}^{A}\right\} \\ +\left(\partial_{z}\bar{\chi}^{B}\right)\left\{\nabla\times\left(\mathbf{e}_{z}\times e^{i\int\beta^{B}dz}\mathbf{E}^{B}\right)+\left(i\omega\mu_{0}\right)\mathbf{e}_{z}\times e^{i\int\beta^{B}dz}\mathbf{H}^{B}\right\} \\ +\bar{\chi}^{A}(z)\left(d_{z}R^{A}\right)\partial_{R^{A}}\left\{\nabla\times\left[\mathbf{e}_{z}\times\left(e^{i\int\beta^{A}dz}\mathbf{E}^{A}\right)\right]+\left(i\omega\mu_{0}\right)\mathbf{e}_{z}\times\left(e^{i\int\beta^{A}dz}\mathbf{H}^{A}\right)\right\} \\ +\bar{\chi}^{B}(z)\left(d_{z}R^{B}\right)\partial_{R^{B}}\left\{\nabla\times\left[\mathbf{e}_{z}\times\left(e^{i\int\beta^{B}dz}\mathbf{E}^{B}\right)\right]+\left(i\omega\mu_{0}\right)\mathbf{e}_{z}\times\left(e^{i\int\beta^{B}dz}\mathbf{H}^{B}\right)\right\} \end{matrix}\right\} \\ \text{ }=\omega^{2}\mu_{0}\varepsilon e^{-i\omega t}\left[\bar{\chi}^{A}(z)e^{i\int\beta^{A}dz}\mathbf{E}^{A}+\bar{\chi}^{B}(z)e^{i\int\beta^{B}dz}\mathbf{E}^{B}\right] \end{matrix}$ (s4)

Applying the integration operation and inner product $\iint dSe^{-i\int\beta^{A}dz}\mathbf{E}^{A*}\cdot$ to both sides of Eq. s4, we can obtain

$\begin{matrix} \text{ }i\left(\frac{d}{dz}\bar{\chi}^{A}\right)4S_{z}^{A}+i\bar{\chi}^{A}(z)\left(\frac{d}{dz}R^{A}\right)\partial_{R^{A}}\left(4S_{z}^{A}\right) \\ =-\omega\left[\iint dS\mathbf{E}^{A*}\cdot\left(\varepsilon-\varepsilon^{A}\right)\mathbf{E}^{A}\right]\bar{\chi}^{A}(z)-\omega e^{i\int\left(\beta^{B}-\beta^{A}\right)dz}\left[\iint dS\mathbf{E}^{A*}\cdot\left(\varepsilon-\varepsilon^{B}\right)\mathbf{E}^{B}\right]\bar{\chi}^{B}(z) \end{matrix}$ (s5a)

$S_{z}^{A}=\frac{1}{4}\iint dS\mathbf{e}_{z}\cdot\left(\mathbf{E}^{A}\times\mathbf{H}^{A*}+\mathbf{E}^{A*}\times\mathbf{H}^{A}\right)$ (s5b)

Letting the net power flow of the fundamental mode of each isolated rod [defined in Eq. s5b] be normalized to 1, Eq. s5a is simplified to

$\text{ }i\frac{d}{dz}\bar{\chi}^{A}=-\kappa^{AA}\bar{\chi}^{A}(z)-e^{i\int\left(\beta^{B}-\beta^{A}\right)dz}\kappa^{AB}\bar{\chi}^{B}(z)$ (s6)

Where, the coefficients$\kappa^{AA}$ and the coupling coefficients $\kappa^{AB}$ are defined as

$\kappa^{AA}=\frac{\omega}{4S_{z}^{A}}\iint dS\mathbf{E}^{A*}\cdot\left(\varepsilon-\varepsilon^{A}\right)\mathbf{E}^{A},\text{ }\kappa^{AB}=\frac{\omega}{4S_{z}^{A}}\iint dS\mathbf{E}^{A*}\cdot\left(\varepsilon-\varepsilon^{B}\right)\mathbf{E}^{B},\text{ }S_{z}^{A}=1$ (s7)

Introducing the new mode coefficient $\chi^{A(B)}(z)$ as

$\chi^{A}(z)=\bar{\chi}^{A}(z)e^{i\frac{1}{2}\int\left(\beta^{A}-\beta^{B}\right)dz},\text{ }\chi^{B}(z)=\bar{\chi}^{B}(z)e^{-i\frac{1}{2}\int\left(\beta^{A}-\beta^{B}\right)dz}$, (s8a)

that is,

$\mathbf{E}=e^{-i\omega t}e^{i\frac{1}{2}\int\left(\beta^{A}+\beta^{B}\right)dz}\left[\chi^{A}(z)\mathbf{E}^{A}\left(R^{A}(z),\mathbf{X}_{\perp}-\mathbf{X}_{0}^{A}\right)+\chi^{B}(z)\mathbf{E}^{B}\left(R^{B}(z),\mathbf{X}_{\perp}-\mathbf{X}_{0}^{B}\right)\right]$, (s8b)

the mode-coefficient equations Eq. s6 of two rods can be rewritten as

$i\frac{d}{dz}\left[\begin{matrix} \chi^{A} \\ \chi^{B} \end{matrix}\right]=\mathbf{H}\left[\begin{matrix} \chi^{A} \\ \chi^{B} \end{matrix}\right]=\left[\begin{matrix} -\frac{1}{2}\left(\beta^{A}-\beta^{B}\right)-\kappa^{AA} & -\kappa^{AB} \\ -\kappa^{BA} & \frac{1}{2}\left(\beta^{A}-\beta^{B}\right)-\kappa^{BB} \end{matrix}\right]\left[\begin{matrix} \chi^{A} \\ \chi^{B} \end{matrix}\right]$. (s9)

Furthermore, in order to guarantee the Hamiltonian ***H*** defined in Eq. s9 to be Hermitian for lossless rods under the first order approximation, we introduce the effective coupling coefficient $\kappa=\sqrt{\left|\kappa^{AB}\kappa^{BA}\right|}$ and neglect the relatively small coefficients$\kappa^{AA}$, $\kappa^{BB}$. Thus, the mode-coefficients equations Eq. s9 of two rods can be approximately simplified as

$i\frac{d}{\kappa dz}\left[\begin{matrix} \chi^{A} \\ \chi^{B} \end{matrix}\right]=i\frac{d}{dZ}\left[\begin{matrix} \chi^{A} \\ \chi^{B} \end{matrix}\right]=\left[\begin{matrix} -\eta& -1 \\ -1 & \eta\end{matrix}\right]\left[\begin{matrix} \chi^{A} \\ \chi^{B} \end{matrix}\right]$. (s10)

Where, $Z=\int\kappa dz,\text{ }\eta=\left(\beta^{A}-\beta^{B}\right)/\left(2\kappa\right)$. Generalizing Eq. s10 to an array of coupled rods arranged in a honeycomb lattice, and only taking the nearest-neighbor coupling into account, we have the following equations for the mode coefficients,

$i\frac{d}{dZ}\chi_{mn}^{A}=-\eta\chi_{mn}^{A}-\chi_{mn}^{B}-\chi_{m\left(n+1\right)}^{B}-\chi_{\left(m-1\right)\left(n+1\right)}^{B}$, (s11a)

$i\frac{d}{dZ}\chi_{mn}^{B}=\eta\chi_{mn}^{B}-\chi_{mn}^{A}-\chi_{m\left(n-1\right)}^{A}-\chi_{\left(m+1\right)\left(n-1\right)}^{A}$. (s11b)

Because the power flow of the fundamental mode of the isolated rod used in Eq. s8b is normalized to 1, for a wave packet propagating in the rod array, the transverse coordinates (*x*, *y*) of its centre of energy (mass) can be defined as

$\mathbf{X}_{\text{C}}=\frac{1}{\sum_{mn} \left(\left|\chi_{mn}^{A}\right|^{2}+\left|\chi_{mn}^{B}\right|^{2}\right)}\sum_{mn} \left[\left|\chi_{mn}^{A}\right|^{2}\left(\mathbf{X}_{0}^{A}+\mathbf{R}_{mn}\right)+\left|\chi_{mn}^{B}\right|^{2}\left(\mathbf{X}_{0}^{B}+\mathbf{R}_{mn}\right)\right]$, (s12)

where, $\mathbf{X}_{0}^{A}=\mathbf{e}_{y}a/\left(2\sqrt{3}\right),\text{ }\mathbf{X}_{0}^{B}=-\mathbf{e}_{y}a/\left(2\sqrt{3}\right)$,$\mathbf{R}_{mn}=\left(m+n/2\right)a\mathbf{e}_{x}+n\left(\sqrt{3}/2\right)a\mathbf{e}_{y}$ is the lattice vector of the array with a lattice constant *a* .

Due to the periodicity in the *x-y* plane, the eigenmodes supported by the periodic rods array are Bloch waves whose mode coefficient $\chi_{mn}^{A(B)}$ can be written as$e^{i\mathbf{k}\cdot\mathbf{R}_{mn}}\chi_{\mathbf{k}}^{A(B)}$, where ***k*** is the Bloch wave vector. Based on Eqs. s11a and s11b, the dynamics of the propagation of Bloch wave along the rods can be cast into the form of Schrödinger-type equation of a two-level system,

$i\frac{d}{dZ}\left[\begin{matrix} \chi_{\mathbf{k}}^{A} \\ \chi_{\mathbf{k}}^{B} \end{matrix}\right]=\mathbf{H}\left(\mathbf{k},\eta\right)\left[\begin{matrix} \chi_{\mathbf{k}}^{A} \\ \chi_{\mathbf{k}}^{B} \end{matrix}\right]=\left[\begin{matrix} -\eta& -S\left(\mathbf{k}\right) \\ -S^{*}\left(\mathbf{k}\right) & \eta\end{matrix}\right]\left[\begin{matrix} \chi_{\mathbf{k}}^{A} \\ \chi_{\mathbf{k}}^{B} \end{matrix}\right]$, (s13a)

$S(\mathbf{k})=1+2\cos\left(\frac{1}{2}k_{x}a\right)e^{\frac{i}{2}\sqrt{3}k_{y}a}$ . (s13b)

By using Pauli matrices$\mathbf{\sigma}_{x}$, $\mathbf{\sigma}_{y}$and$\mathbf{\sigma}_{z}$, Eq. s13a can be written as Eq. 1 in the main text with$|\mathbf{u}_{\mathbf{k}}\rangle=\left[\begin{matrix} \chi_{\mathbf{k}}^{A} & \chi_{\mathbf{k}}^{B} \end{matrix}\right]^{T}$,

$i\frac{d}{dZ}|\mathbf{u}_{\mathbf{k}}\rangle=\mathbf{H}\left(\mathbf{k},\eta\right)|\mathbf{u}_{\mathbf{k}}\rangle$, $\mathbf{H}\left(\mathbf{k},\eta\right)=-\mathrm{Re}\left[S(\mathbf{k})\right]\mathbf{\sigma}_{x}+\mathrm{Im}\left[S(\mathbf{k})\right]\mathbf{\sigma}_{y}-\eta\mathbf{\sigma}_{z}$. (1)

**Note 2. The detailed derivation of the velocity equations of Bloch wave propagating along the rods array [Eq. 5 in the main text]**

The mode-coefficient equations for the array of rods (Eq. s11a and s11b) can be rewritten in full matrix form as,

$i\frac{d}{dZ}\left[\begin{matrix} \left[\chi_{mn}^{A}\right] \\ \left[\chi_{mn}^{B}\right] \end{matrix}\right]=\mathbf{H}\left[\begin{matrix} \left[\chi_{mn}^{A}\right] \\ \left[\chi_{mn}^{B}\right] \end{matrix}\right]=\left[\begin{matrix} \mathbf{H}^{AA} & \mathbf{H}^{AB} \\ \mathbf{H}^{BA} & \mathbf{H}^{BB} \end{matrix}\right]\left[\begin{matrix} \left[\chi_{mn}^{A}\right] \\ \left[\chi_{mn}^{B}\right] \end{matrix}\right]$, (s14)

where,$H_{(mn)(pq)}^{AA}=-\eta\delta_{m,p}\delta_{n,q}=-H_{(mn)(pq)}^{BB},\text{ }$ $H_{(mn)(pq)}^{AB}=-\left(\delta_{m,p}\delta_{n,q}+\delta_{m,p}\delta_{n+1,q}+\delta_{m-1,p}\delta_{n+1,q}\right)$, and$\mathbf{H}^{BA}=\left(\mathbf{H}^{AB}\right)^{T}$. Combining the mode coefficient equations with the definition of the transverse coordinate of the centre of energy (Eq. s12), we can obtain the centre-of-energy velocity of a wave packet propagating in the array of rods

$\frac{d\mathbf{X}_{\text{C}}}{dZ}=\frac{1}{\sum_{mn} \left(\left|\chi_{mn}^{A}\right|^{2}+\left|\chi_{mn}^{B}\right|^{2}\right)}\left\{\begin{matrix} i\langle\left[\begin{matrix} \left[\chi_{mn}^{A}\right] \\ \left[\chi_{mn}^{B}\right] \end{matrix}\right]|\left[\begin{matrix} \left(\mathbf{H}^{AA}\bar{\mathbf{R}}-\bar{\mathbf{R}}\mathbf{H}^{AA}\right) & \left(\mathbf{H}^{AB}\bar{\mathbf{R}}-\bar{\mathbf{R}}\mathbf{H}^{AB}\right) \\ \left(\mathbf{H}^{BA}\bar{\mathbf{R}}-\bar{\mathbf{R}}\mathbf{H}^{BA}\right) & \left(\mathbf{H}^{BB}\bar{\mathbf{R}}-\bar{\mathbf{R}}\mathbf{H}^{BB}\right) \end{matrix}\right]|\left[\begin{matrix} \left[\chi_{mn}^{A}\right] \\ \left[\chi_{mn}^{B}\right] \end{matrix}\right]\rangle\\ +i\left(\mathbf{X}_{0}^{B}-\mathbf{X}_{0}^{A}\right)\langle\left[\begin{matrix} \left[\chi_{mn}^{A}\right] \\ \left[\chi_{mn}^{B}\right] \end{matrix}\right]|\left[\begin{matrix} 0 & \mathbf{H}^{AB} \\ -\mathbf{H}^{BA} & 0 \end{matrix}\right]|\left[\begin{matrix} \left[\chi_{mn}^{A}\right] \\ \left[\chi_{mn}^{B}\right] \end{matrix}\right]\rangle\end{matrix}\right\}$(s15)

in which, $\bar{\mathbf{R}}_{(mn)(pq)}=R_{mn}\delta_{m,p}\delta_{n,q}$. For Bloch waves with the mode coefficient$\chi_{mn}^{A(B)}$=$e^{i\mathbf{k}\cdot\mathbf{R}_{mn}}\chi_{\mathbf{k}}^{A(B)}$, using the Hamiltonian defined in Eq. s13a (Eq. 1), the equation of motion (Eq. s15) can be simplified as

$\frac{d\mathbf{X}_{\text{C}\mathbf{k}}}{dZ}=\frac{1}{\left|\chi_{\mathbf{k}}^{A}\right|^{2}+\left|\chi_{\mathbf{k}}^{B}\right|^{2}}\left[\begin{matrix} \text{ }\langle\left[\begin{matrix} \chi_{\mathbf{k}}^{A} \\ \chi_{\mathbf{k}}^{B} \end{matrix}\right]|\partial_{\mathbf{k}}\mathbf{H}\left(\mathbf{k},\eta\right)|\left[\begin{matrix} \chi_{\mathbf{k}}^{A} \\ \chi_{\mathbf{k}}^{B} \end{matrix}\right]\rangle\\ +i\left(\mathbf{X}_{0}^{B}-\mathbf{X}_{0}^{A}\right)\langle\left[\begin{matrix} \chi_{\mathbf{k}}^{A} \\ \chi_{\mathbf{k}}^{B} \end{matrix}\right]|\left[\begin{matrix} 0 & -S(\mathbf{k}) \\ S^{*}(\mathbf{k}) & 0 \end{matrix}\right]|\left[\begin{matrix} \chi_{\mathbf{k}}^{A} \\ \chi_{\mathbf{k}}^{B} \end{matrix}\right]\rangle\end{matrix}\right]$ (s16)

Using$|\mathbf{u}_{\mathbf{k}}\rangle=\left[\begin{matrix} \chi_{\mathbf{k}}^{A} & \chi_{\mathbf{k}}^{B} \end{matrix}\right]^{T}$, Eq. s16 can be written as Eq. 5 in the main text.

$\frac{d\mathbf{X}_{\text{C}\mathbf{k}}}{dZ}=\frac{1}{\langle\mathbf{u}_{\mathbf{k}}|\mathbf{u}_{\mathbf{k}}\rangle}\langle\mathbf{u}_{\mathbf{k}}|\partial_{\mathbf{k}}\mathbf{H}\left(\mathbf{k},\eta\right)|\mathbf{u}_{\mathbf{k}}\rangle+\frac{\left(\mathbf{X}_{0}^{B}-\mathbf{X}_{0}^{A}\right)}{\langle\mathbf{u}_{\mathbf{k}}|\mathbf{u}_{\mathbf{k}}\rangle}\left[\langle\mathbf{u}_{\mathbf{k}}|\mathrm{Im}\left[S(\mathbf{k})\right]\mathbf{\sigma}_{x}+\mathrm{Re}\left[S(\mathbf{k})\right]\mathbf{\sigma}_{y}|\mathbf{u}_{\mathbf{k}}\rangle\right]$ (5)

**Note 3. The detailed derivation of the** **mode-coefficient equations** (**Eq. 6a)** **and the velocity equations (Eq. 6b in the main text)**

Without loss of generality, the lower- (***Ψ***_1_) and upper- (***Ψ***_2_)-energy states of Hamiltonian ***H*(*k*,** *η***)** in Eq. 1 with the eigen values equal to $-\beta_{Z}$ and $\beta_{Z}=\sqrt{\eta^{2}+\left|S\left(\mathbf{k}\right)\right|^{2}}$, respectively, can be selected to have the following form

$|\mathbf{\psi}_{1}\rangle=\frac{1}{\sqrt{\left|\beta_{Z}+\eta\right|^{2}+\left|S(\mathbf{k})\right|^{2}}}\left[\begin{matrix} \beta_{Z}+\eta\\ S^{*}(\mathbf{k}) \end{matrix}\right],\text{ }|\mathbf{\psi}_{2}\rangle=\frac{1}{\sqrt{\left|\beta_{Z}+\eta\right|^{2}+\left|S(\mathbf{k})\right|^{2}}}\left[\begin{matrix} -S(\mathbf{k}) \\ \beta_{Z}+\eta\end{matrix}\right]$, (s17)

Expressing the Bloch wave in Eq. 1 as a superposition of the two eigenstates,

$|\mathbf{u}_{\mathbf{k}}\rangle=b_{1}(Z)|\mathbf{\psi}_{1}\rangle+b_{2}(Z)|\mathbf{\psi}_{2}\rangle=\left[|\mathbf{\psi}_{1}\rangle,|\mathbf{\psi}_{2}\rangle\right]\mathbf{\rho},\text{ }$ $\text{ }\mathbf{\rho}=\left[b_{1},b_{2}\right]^{T}$ (s18)

we can directly obtain the mode-coefficient equations (Eq. 6a in the main text) from Eq. 1,

$i\frac{d}{dZ}\mathbf{\rho}=\left[\bar{\mathbf{H}}_{\mathbf{k}}-\mathbf{\Lambda}^{(\eta)}\eta'\right]\mathbf{\rho}.$ (6a)

where, $\bar{\mathbf{H}}_{\mathbf{k}}=\text{diag}[-\beta_{Z},\beta_{Z}]$, $\eta'=d\eta/dZ,$ $\mathbf{\Lambda}_{12}^{(\eta)}=i\langle\mathbf{\psi}_{1}|\partial_{\eta}\mathbf{\psi}_{2}\rangle=\mathbf{\Lambda}_{21}^{(\eta)*},\text{ }\mathbf{\Lambda}_{11}^{(\eta)}=\mathbf{\Lambda}_{22}^{(\eta)}=0$.

For the normalized Bloch wave$\langle\mathbf{u}_{\mathbf{k}}|\mathbf{u}_{\mathbf{k}}\rangle=1$, the velocity equation (Eq. 5) can be rewritten as

$\frac{d\mathbf{X}_{\text{C}\mathbf{k}}}{dZ}=\partial_{\mathbf{k}}\langle\mathbf{u}_{\mathbf{k}}|\mathbf{H}\left(\mathbf{k},\eta\right)|\mathbf{u}_{\mathbf{k}}\rangle-\langle\partial_{\mathbf{k}}\mathbf{u}_{\mathbf{k}}|\mathbf{H}\left(\mathbf{k},\eta\right)|\mathbf{u}_{\mathbf{k}}\rangle-\langle\mathbf{u}_{\mathbf{k}}|\mathbf{H}\left(\mathbf{k},\eta\right)|\partial_{\mathbf{k}}\mathbf{u}_{\mathbf{k}}\rangle+\left(\mathbf{X}_{0}^{B}-\mathbf{X}_{0}^{A}\right)\langle\mathbf{u}_{\mathbf{k}}|\mathbf{\Xi}(\mathbf{k})|\mathbf{u}_{\mathbf{k}}\rangle$(s19)

in which, $\text{ }\mathbf{\Xi}(\mathbf{k})=\mathrm{Im}\left[S(\mathbf{k})\right]\mathbf{\sigma}_{x}+\mathrm{Re}\left[S(\mathbf{k})\right]\mathbf{\sigma}_{y}$. Combining Eq. s17, Eq. s18 and Eq. s19, we have

$\frac{d\mathbf{X}_{\text{C}\mathbf{k}}}{dZ}=\mathbf{\rho}^{+}\left(\partial_{\mathbf{k}}\bar{\mathbf{H}}_{\mathbf{k}}\right)\mathbf{\rho}-i\mathbf{\rho}^{+}\mathbf{\Lambda}^{(\mathbf{k})}\bar{\mathbf{H}}_{\mathbf{k}}\mathbf{\rho}+i\mathbf{\rho}^{+}\bar{\mathbf{H}}_{\mathbf{k}}\mathbf{\Lambda}^{(\mathbf{k})}\mathbf{\rho}+\left(\mathbf{X}_{0}^{B}-\mathbf{X}_{0}^{A}\right)\mathbf{\rho}^{+}\mathbf{\Xi}(\mathbf{k})\mathbf{\rho}$. (s20)

While, Eq. 6a can be transformed as

$\bar{\mathbf{H}}_{\mathbf{k}}\mathbf{\rho}=i\frac{d}{dZ}\mathbf{\rho}+\mathbf{\Lambda}^{(\eta)}\mathbf{\rho}\eta',\text{ }\mathbf{\rho}^{+}\bar{\mathbf{H}}_{\mathbf{k}}=-i\frac{d}{dZ}\mathbf{\rho}^{+}+\mathbf{\rho}^{+}\mathbf{\Lambda}^{(\eta)}\eta'$. (s21)

Thus, Eq. s20 can be expressed as

$\begin{matrix} \frac{d\mathbf{X}_{\text{C}\mathbf{k}}}{dZ}=\mathbf{\rho}^{+}\left(\partial_{\mathbf{k}}\bar{\mathbf{H}}_{\mathbf{k}}\right)\mathbf{\rho}-\mathbf{\rho}^{+}i\left[\mathbf{\Lambda}^{(\mathbf{k})}\mathbf{\Lambda}^{(\eta)}-\mathbf{\Lambda}^{(\eta)}\mathbf{\Lambda}^{(\mathbf{k})}\right]\mathbf{\rho}\eta' \\ \text{ }+\frac{d}{dZ}\left(\mathbf{\rho}^{+}\mathbf{\Lambda}^{(\mathbf{k})}\mathbf{\rho}\right)-\mathbf{\rho}^{+}\left(\frac{d}{dZ}\mathbf{\Lambda}^{(\mathbf{k})}\right)\mathbf{\rho}+\left(\mathbf{X}_{0}^{B}-\mathbf{X}_{0}^{A}\right)\mathbf{\rho}^{+}\left[\mathbf{\Xi}(\mathbf{k})\right]\mathbf{\rho} \end{matrix}$ (s22)

Introducing to denote the state average, e.g.$\left\langle\mathbf{\Lambda}^{(\mathbf{k})}\right\rangle=\mathbf{\rho}^{+}\mathbf{\Lambda}^{(\mathbf{k})}\mathbf{\rho}$, Eq. s22 can be rewritten as Eq. 6b in the main text. That is

$\frac{d}{dZ}\left[\mathbf{X}_{\text{C}\mathbf{k}}-\left\langle\mathbf{\Lambda}^{(\mathbf{k})}\right\rangle\right]=\left\langle\partial_{\mathbf{k}}\bar{\mathbf{H}}_{\mathbf{k}}\right\rangle-\left\langle\mathbf{\Omega}^{(\mathbf{k}\eta)}\right\rangle\eta'+\left(\mathbf{X}_{0}^{B}-\mathbf{X}_{0}^{A}\right)\left\langle\mathbf{\Xi}(\mathbf{k})\right\rangle-\left\langle\frac{d}{dZ}\mathbf{\Lambda}^{(\mathbf{k})}\right\rangle$. (6b)

Where, Berry curvature $\text{ }\mathbf{\Omega}^{(\mathbf{k}\eta)}=i\left[\mathbf{\Lambda}^{(\mathbf{k})}\mathbf{\Lambda}^{(\eta)}-\mathbf{\Lambda}^{(\eta)}\mathbf{\Lambda}^{(\mathbf{k})}\right]$ is defined in the synthetic space [***k*,** *η*].

**Note 4. The details of the method used to rigorously simulate the propagation of Gaussian beam in the rods array**

We simulate the propagation of Gaussian beam in the proposed rod array based on Eqs. s11a and s11b, and s12. For an incident Gaussian beam with a centre of energy ***X*_0_**, central wave-vector ***k***_C_ and beam waist radius *W*, the initial values of modes coefficients in Eqs. s11a and s11b are given by

$\chi_{mn}^{A(B)}(Z_{0})=\alpha^{A(B)}\psi\left(\mathbf{X}_{0}^{A(B)}+\mathbf{R}_{mn}\right)$, $\psi\left(\mathbf{X}\right)=\frac{1}{2\pi}e^{i\mathbf{k}_{C}\cdot\left(\mathbf{X}-\mathbf{X}_{0}\right)}\exp\left[\frac{-\left|\mathbf{X}-\mathbf{X}_{0}\right|^{2}}{2W^{2}}\right]$. (s23)

The coefficients (*ɑ^A^, ɑ^B^*) in Eq. s23 are equal to $(1,1)/\sqrt{2}$ when the input beam on the sub-lattices A and B are identical at the position *Z*_0_. For the input beam in the lower-energy state, the coefficients are

$\left[\begin{matrix} \alpha^{A} \\ \alpha^{B} \end{matrix}\right]=|\mathbf{\Psi}_{1}\rangle=\frac{1}{\sqrt{\left|\beta_{z}+\eta\right|^{2}+\left|S(\mathbf{k})\right|^{2}}}\left[\begin{matrix} \beta_{z}+\eta\\ S^{*}(\mathbf{k}) \end{matrix}\right]$ (s24)

The dots in the Fig. s1a and s1b show the calculated centre-of-energy Hall shifts and 3D space trajectory of the Gaussian beam with parameters (***X*_0_, *k***_C_, *W*)=$\left(0,0.8\mathbf{K},50/\left|\mathbf{K}\right|\right)$ propagating in the hexagonal array. They agree well with those of the Bloch wave with wave vector ***k*** equal to ***k***_C_ obtained by solving Eqs. 6a and 6b which are the solid lines in Fig. s1a and s1b. For the incident Gaussian beam with (***X*_0_, *k***_C_, *W*) =$\left(0,0.95\mathbf{K},50/\left|\mathbf{K}\right|\right)$**,** Fig. s1c and s1d present the similar results.


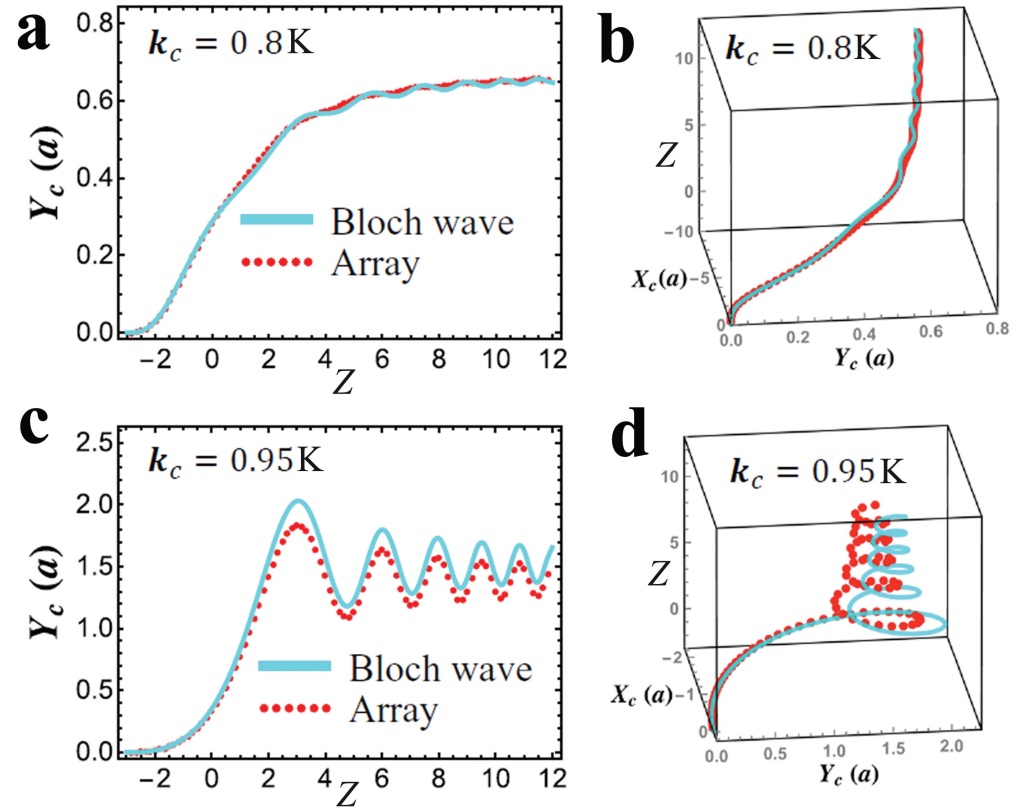


**FIG. s1** Evolutions of the centre of energy when the Gaussian beams propagating in the proposed coupled rods array. For the incident Gaussian beam with the central wave-vector ***k***_c_ = 0.8**K** in the lower-energy state，**a** and **b** show the Hall shift and spatial trajectory of the Gauss-beam centre. The dots are the results by simulating the propagation of Gaussian beam in the rods array. While, the solid lines are those of Bloch wave obtained by solving Eqs.(6a) and (6b). **c** and **d** Corresponding results of the same Gaussian beam with ***k***_c_ = 0.95**K**.

Taking into account the beam waist radius of Gaussian beams, we can average the transverse shift and spatial trajectory of Bloch waves (Eq. 6b) over the incident Gaussian beam in the space of momentum, which is defined as

$\bar{\mathbf{X}}_{C}=\frac{\iint dk_{x}dk_{y}\left|g\left(k_{x},k_{y}\right)\right|^{2}\mathbf{X}_{\text{C}\mathbf{k}}}{\iint dk_{x}dk_{y}\left|g\left(k_{x},k_{y}\right)\right|^{2}}$, $g\left(k_{x},k_{y}\right)=\iint dxdye^{-i\left(k_{x}x+k_{y}y\right)}\psi\left(\mathbf{X}\right)$. (s25)

Where, g(*k*_x_, *k*_y_) is the distribution of the incident Gaussian beam in the ***k*** space. Figure s2 shows that the averaged results agree better with those obtained by the rigorous simulation than the non-averaged results shown in Fig. s1.


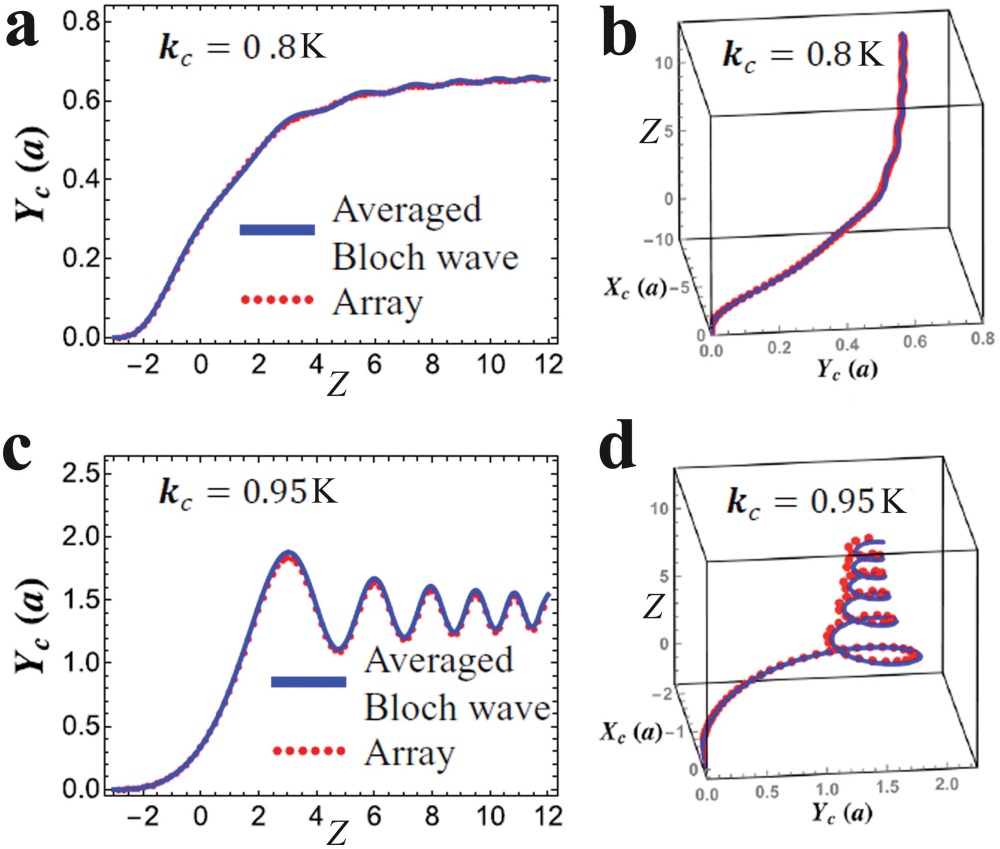


**FIG. s2** Identical parameters as in Fig. s1, but solid lines are now calculated by averaging results for incident Bloch waves with different wave vectors.

**Note 5. The method used to calculate the effective coupling coefficient**

To obtain the effective coupling coefficient of rods arranged in the honeycomb lattice, we consider the coupling between the four nearest-neighbor-coupled rods with invariant radii shown in Fig. s3. With the rod A_mn_ in the sub-lattice A located at the centre (Fig. s3a), based on the mode coefficient equations Eq. s11a and Eq. s11b, we have

$i\frac{d}{dZ}\left[\begin{matrix} \chi_{mn}^{A} \\ \chi_{mn}^{B} \\ \chi_{m\left(n+1\right)}^{B} \\ \chi_{\left(m-1\right)\left(n+1\right)}^{B} \end{matrix}\right]=\left[\begin{matrix} -\eta& -1 & -1 & -1 \\ -1 & \eta& 0 & 0 \\ -1 & 0 & \eta& 0 \\ -1 & 0 & 0 & \eta\end{matrix}\right]\left[\begin{matrix} \chi_{mn}^{A} \\ \chi_{mn}^{B} \\ \chi_{m\left(n+1\right)}^{B} \\ \chi_{\left(m-1\right)\left(n+1\right)}^{B} \end{matrix}\right]=-\left(\frac{\bar{\beta}^{A}}{\kappa}-\frac{\beta^{A}+\beta^{B}}{2\kappa}\right)\left[\begin{matrix} \chi_{mn}^{A} \\ \chi_{mn}^{B} \\ \chi_{m\left(n+1\right)}^{B} \\ \chi_{\left(m-1\right)\left(n+1\right)}^{B} \end{matrix}\right]$ (s26)

Where, $\bar{\beta}^{A}$is the propagation constant of the eigen mode supported by the four rods with eigen-fields mainly located at the rod A_mn_ , which can be obtained by the COMSOL simulation. Thus, we can get the coupling coefficient from Eq. s26,

$\kappa=\sqrt{\frac{1}{3}\left(\bar{\beta}^{A}-\beta^{B}\right)\left(\bar{\beta}^{A}-\beta^{A}\right)}$ (s27)

Similarly, we can obtain the propagation constant $\bar{\beta}^{B}$of the four rods shown in Fig. s3b. Hence, using Eq. s27, we can calculate the effective coupling coefficient of the nearest-neighbor rods in sub-lattices A and B of the honeycomb lattice by the following formula

$\kappa=\frac{1}{2}\left(\sqrt{\frac{1}{3}\left(\bar{\beta}^{A}-\beta^{B}\right)\left(\bar{\beta}^{A}-\beta^{A}\right)}+\sqrt{\frac{1}{3}\left(\bar{\beta}^{B}-\beta^{B}\right)\left(\bar{\beta}^{B}-\beta^{A}\right)}\right)$ (s28)

**
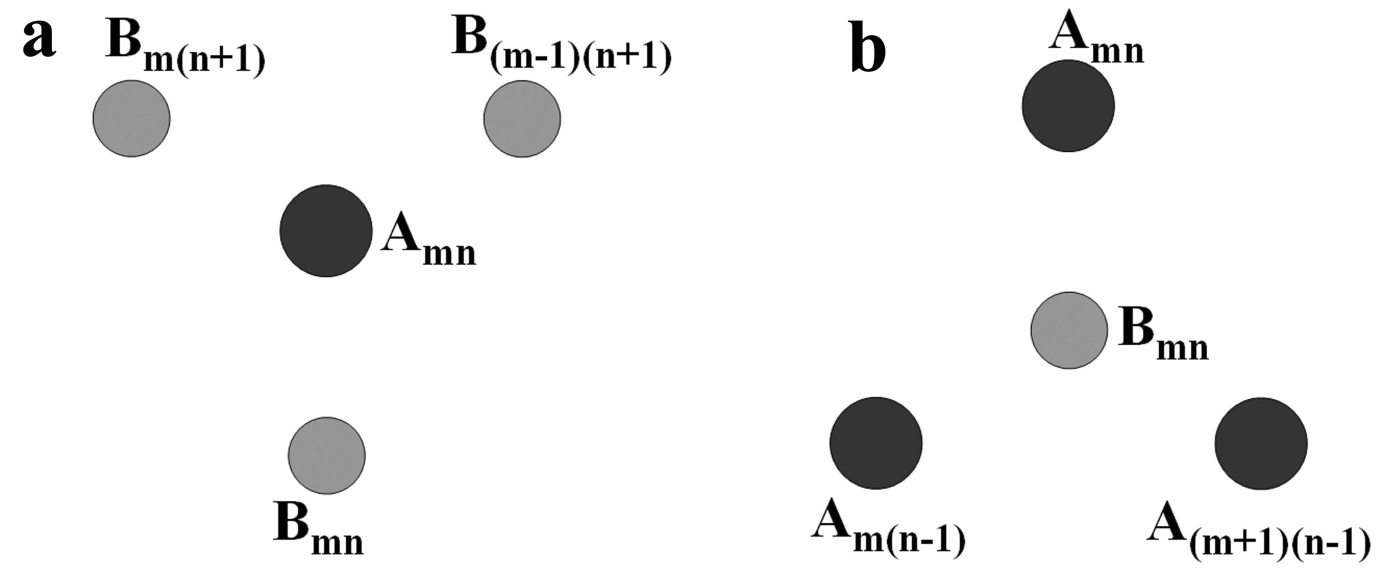
**

**FIG. s3** Schematic illustration of the four nearest-neighbor-coupled rods in a honeycomb lattice. **a** The rod in the sub-lattice A is located at the centre. **b** The rod in the sub-lattice B is located at the centre.
